# Global, regional, and national childhood brain and central nervous system cancer burden: an analysis based on the Global Burden of Disease Study

**DOI:** 10.1186/s41182-025-00810-9

**Published:** 2025-10-02

**Authors:** Zhenjin Su, Jie Lu, Yuheng Shi, Tian Li, Bin Qi, Zeshang Guo

**Affiliations:** 1 Department of Neurosurgery, The First Hospital of Jilin University, Changchun, 130000 Jilin Province China; 2 Department of Pediatric, The First Hospital of Jilin University, Changchun, 130000 Jilin Province China; 3 Tianjin Key Laboratory of Acute Abdomen Disease-Associated Organ Injury and ITCWM Repair, Institute of Integrative Medicine of Acute Abdominal Diseases, Tianjin Nankai Hospital, Tianjin Medical University, 8 Changjiang Avenue, Tianjin, 300100 China

**Keywords:** Epidemiology, Central nervous system cancer, Children, Global burden of disease, Prediction

## Abstract

**Objectives:**

We assessed the global, regional, and national burdens of childhood brain and central nervous system cancer from 1990–2021 (the latest year).

**Methods:**

We utilized data from the 2021 Global Burden of Disease Study and analyzed trends in childhood brain and central nervous system cancers through joinpoint regression. We assessed the global burden of childhood brain and central nervous system cancers from various perspectives. Finally, the Bayesian age‒period‒cohort model was employed to forecast future trends through 2030.

**Results:**

Childhood brain and CNS cancers are the most common solid tumors and the leading cause of death in children. From 1990 to 2021, the age-standardized incidence, prevalence, mortality, and DALYs decreased. The incidence is slightly greater in boys than in girls and peaks at 0–4 years of age, decreasing with age. The disease burden correlates with sociodemographic indices, with higher burdens observed in regions with higher sociodemographic indices. Future projections indicate a continued decline in incidence, prevalence, mortality, and DALYs.

**Conclusions:**

While the global burden of childhood brain and CNS cancer has significantly decreased due to medical advancements, childhood cancer continues to be a major cause of childhood mortality. Further optimization of global health resources is crucial for alleviating this burden.

**Graphical Abstract:**

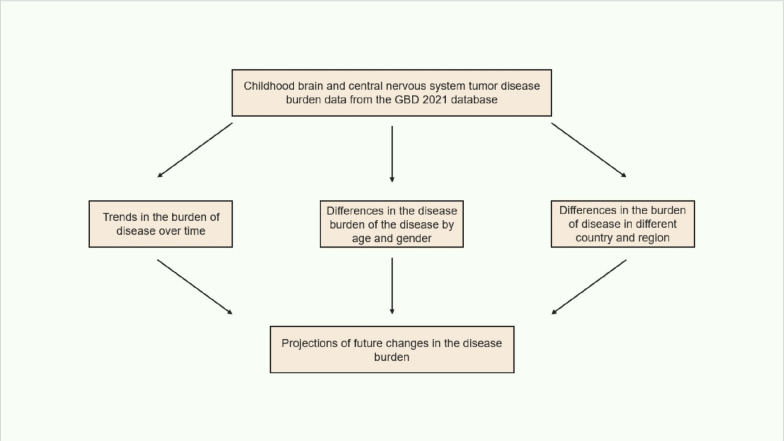

**Supplementary Information:**

The online version contains supplementary material available at 10.1186/s41182-025-00810-9.

## Introduction

Neoplasms remain the main killer worldwide [1–4]. Childhood brain and central nervous system (CNS) cancers are the most common solid tumors in children and the leading cause of childhood mortality, particularly in developed countries [5–8]. These cancers are often diagnosed late and are associated with a poor prognosis [9, 10].

Brain and CNS cancers encompass tumors of the brain and spinal cord, with brain tumors constituting more than 90% of cases [11]. Research indicates that the incidence of these cancers in children decreases with age, with the highest incidence occurring in the 0–4 year age group [5, 12, 13]. Malignant tumors constitute approximately 30% of childhood brain and CNS cancers. Gliomas and germ cell tumors are more common in boys, whereas pituitary tumors and meningiomas are more prevalent in girls [11]. Brain and CNS cancers typically present with nonspecific symptoms, such as headaches and dizziness. In children, these symptoms often manifest as poor learning and fatigue. The nonspecific nature of these symptoms often leads to the disease being overlooked [14]. Despite recent improvements in prognosis due to advances in medical science, updated therapeutic guidelines, and targeted molecular therapies, childhood brain and CNS cancers continue to represent a substantial global health challenge [15–19]. Particularly in regions with a low sociodemographic index (SDI), the disease burden is significantly exacerbated due to inadequate diagnosis, limited treatment options, and a lack of effective management for posttreatment complications [20–22].

**Fig. 1:**
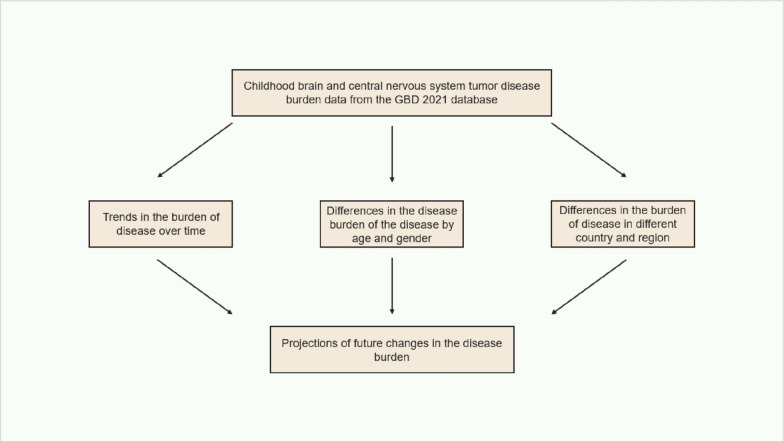
Graphical abstract

Therefore, it is crucial to assess the specific burden of childhood brain and CNS cancers to inform efforts to mitigate this global health issue. The Global Burden of Disease Study (GBD) 2021 (the latest year) provides four key metrics to assess the burden, covering data from 204 countries and territories across various regions [23]. As shown in Fig. 1, using the latest GBD 2021 data, we analyzed the burden and trends of childhood brain and CNS cancers at the global, regional, national, and local levels from 1990 to 2021.

## Method

### Study population

Brain and CNS cancer data, as defined by the GBD project, were collected for both sexes across three age categories (< 5 years, 5–9 years, 10–14 years) and 204 national and regional subcategories. We categorized children as 0–14 years old and divided them into three age subcategories, namely, < 5 years, 5–9 years, and 10–14 years, to better analyze age-related differences. All the countries and territories were categorized into 21 regions on the basis of their epidemiological similarities and geographical proximity.

### Data collection

We analyzed data from the Global Burden of Disease (GBD) Study 2021 (https://ghdx.healthdata.org/gbd-2021/sources). This dataset includes information on 369 diseases and injuries, including brain and CNS cancer, across 204 countries and regions from 1990 to 2021. In this study, we extracted data on the incidence, prevalence, mortality and disability-adjusted life years (DALYs) of brain and central nervous system cancers in individuals aged 0–14 years from the GBD 2021 through the GBD Results Tool (https://vizhub.healthdata.org/gbdresults/).

Incident cases, prevalent cases, mortality, disability-adjusted life years, incidence rates, and prevalence rates were extracted directly from GBD 2021, with all rates reported per 100,000 people. The 95% uncertainty interval (UI) was determined by the 25th and 95th percentiles of the 1000 estimates generated by the GBD algorithm.

### Sociodemographic index

The GBD 2021 also provides the social demographic index (SDI) for each country, a composite measure reflecting social and economic conditions that influence health outcomes. The SDI is calculated as the geometric mean of three indices (scaled from 0 to 1): total fertility rate among individuals under 25, average years of education for individuals aged 15 and older, and lag-distributed income per capita. An SDI of 0 indicates minimal education, low per capita income, and high fertility rates. The SDI is divided into five quintiles: low, lower-middle, middle, upper-middle, and high regions.

### Statistical analysis

We calculated age-standardized rates (ASRs) per 100,000 people with brain or CNS cancer from 0 to 14 years of age via the following formula:

$$\frac{{\Sigma }_{i=1}^{N}{\alpha }_{i}{W}_{i}}{{\Sigma }_{i=1}^{N}{W}_{i}}$$

In the equation, ${\alpha }_{i}$ represents the age-specific rate in the $i$ th age group, whereas ${W}_{i}$ denotes the count of individuals within the same age group on the basis of the GBD 2021 standard population.$N$ is the total number of age categories.

The primary objective of this study was to analyze global trends in the incidence, prevalence, mortality, and DALYs associated with childhood brain and CNS cancer. We computed the average annual percentage change (AAPC) for these metrics across the 0–14 age group and its three subcategories via linear regression models. The AAPC summarizes trends over a specified interval, computed as a weighted average of annual percentage change (APC), allowing a single value to represent the average trend over multiple years. The AAPC was determined via the geometrically weighted average of the APCs. The AAPC value represents the annual rate of variation; for example, an AAPC of 0.5 indicates a yearly increase of 0.5%.

The second aim was to determine periods with significant changes in the incidence, prevalence, mortality, and DALY trends for childhood brain and CNS cancer. To achieve this goal, we applied joinpoint regression analysis to detect temporal changes and fit the most parsimonious model by connecting separate line segments on a logarithmic scale. These portions, known as joinpoints, mark changes in trend. The final model is based on professional judgment and the weighted Bayesian information criterion within the joinpoint software. We then examined trends in incidence, prevalence, mortality, and DALYs by age, sex, five SDI regions, 21 GBD regions, and 204 countries. Tendencies were plotted by the SDI across the 21 GBD regions and 204 countries. Finally, we projected the incidence, prevalence, mortality, and DALYs of childhood brain and CNS cancer for 2030 projections via the Bayesian age‒period‒cohort model.

All the statistical analyses were performed via RStudio (version 2024.4.2.0) and the joinpoint regression program (version 4.9.1.0).

## Results

### Age-standardized global trends

**Fig. 2:**
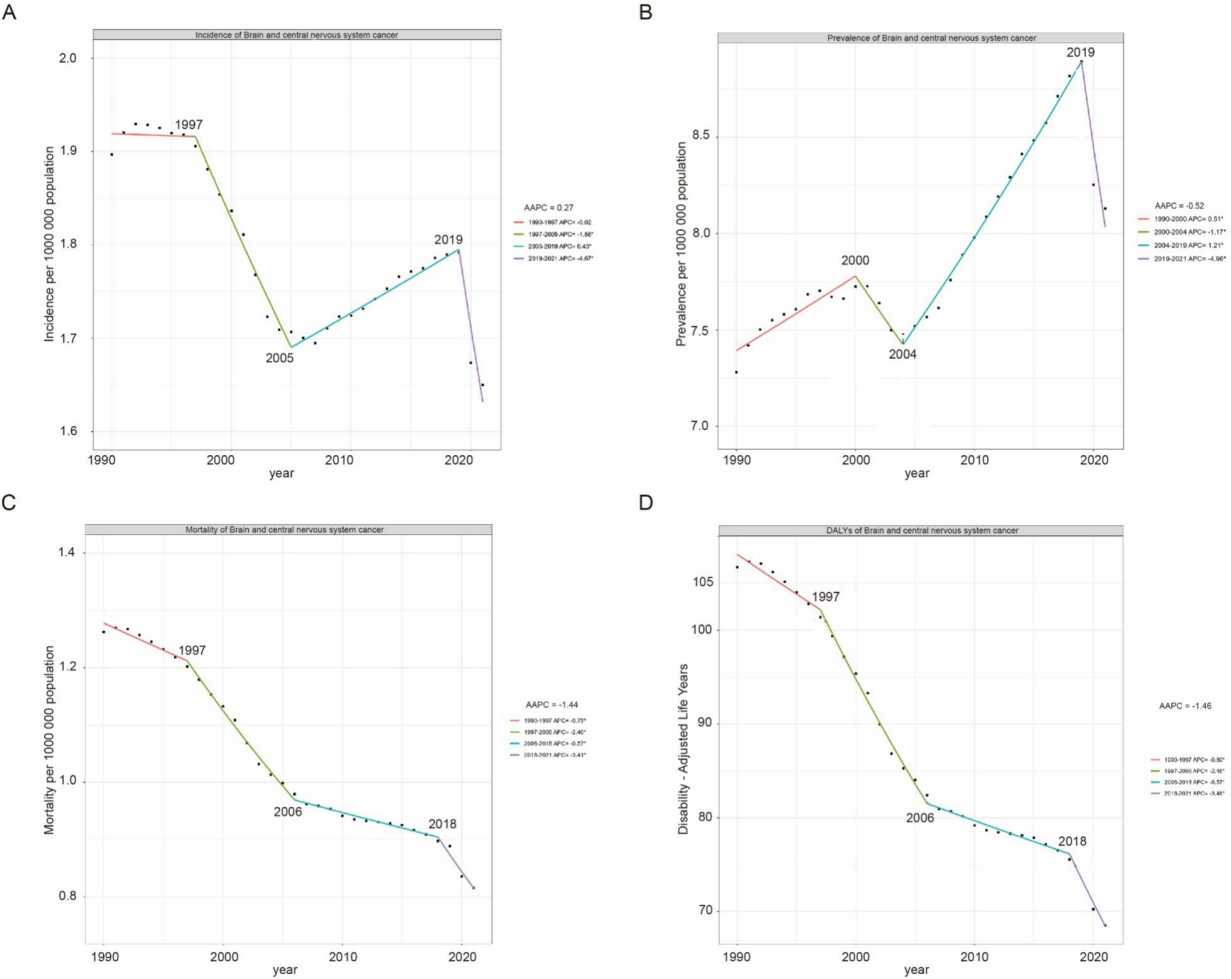
Global trends in age-standardized rates (per 100,000 people) of childhood brain and central nervous system cancers from 1990 to 2021. **A** Age-standardized incidence; **B** age-standardized incidence; **C** age-standardized mortality; **D** age-standardized DALYs

Globally, the incidence of childhood brain and CNS cancer generally declined from 1990 to 2021 (AAPC = − 0.52, 95% CI [− 0.62, − 0.42]). Joinpoint regression analysis revealed marked shifts in incidence rates during 1997, 2005, and 2019. The incidence rate experienced a slight decline from 1990 to 1997 (AAPC = − 0.02, 95% UI [− 0.22, 0.17]), a marked decline between 1998 and 2005 (AAPC = − 1.56, 95% UI [− 1.72, − 1.39]), an increase from 2006 to 2019 (AAPC = 0.43, 95% UI [0.37, 0.49]), and a significant decline after 2020 (AAPC = − 4.67, 95% UI [− 5.90, − 3.41]). Joinpoint regression also revealed marked shifts in disease incidence in 2000, 2004, and 2019. While there was a decline in prevalence between 2000 and 2004 and post-2019 (AAPC = − 1.17, 95% UI [− 1.89, − 0.44]; AAPC = − 4.96, 95% UI [− 6.61, − 3.29]), the overall trend from 1990 to 2021 was an increase (AAPC = 0.27, 95% CI [0.12, 0.42]). As shown in Fig. 2 and Tables 1, 2.

**Table 1:** Global burden of childhood cancer in 2021 and its AAPC from 1990 to 2021

	Incidence				Prevalence			
	Cases (n), 1990	Cases (n), 2021	AAPC, 1990–2021	p value	Cases (n), 1990	Cases (n), 2021	AAPC, 1990–2021	p value
Global	33,107 (25,238 to 41,603)	33,091 (26,727 to 40,701)	− 0.52 (− 0.62 to − 0.42)	< 0.001	127,138 (99,975 to 155,213)	162,879 (133,143 to 199,896)	0.27 (0.12 to 0.42)	0.001
sex								
Male	18,166 (11,497 to 25,766)	17,255 (11,733 to 23,502)	− 0.68 (− 0.82 to − 0.53)	< 0.001	68,068 (45,563 to 99,223)	84,536 (57,733 to 113,772)	0.19 (0.02 to 0.35)	0.026
Female	14,942 (10,289 to 21,897)	15,836 (13,158 to 18,474)	− 0.32 (− 0.42 to − 0.22)	< 0.001	59,070 (42,573 to 82,199)	78,343 (64,131 to 92,352)	0.38 (0.25 to 0.52)	< 0.001
Age group, years								
0–4	15,340 (11,231 to 20,140)	11,617 (8947 to 14,807)	− 1.2 (− 1.32 to − 1.07)	< 0.001	61,472 (46,312 to 78,413)	59,475 (46,057 to 75,035)	− 0.42 (− 0.67 to − 0.17)	0.001
5–9	10,464 (8138 to 12,932)	11,509 (9336 to 13,927)	− 0.26 (− 0.45 to − 0.06)	0.009	37,752 (30,360 to 45,216)	53,661 (44,498 to 64,794)	0.58 (0.34 to 0.81)	< 0.001
10–14	7304 (5869 to 8531)	9965 (8444 to 11,967)	0.29 (0.14 to 0.45)	< 0.001	27,915 (23,302 to 31,584)	49,743 (42,589 to 60,067)	1.17 (1.00 to 1.35)	< 0.001

**Table 2:** The mortality and DALYs of childhood cancer in 2021 and its AAPC from 1990 to 2021

	Mortality				DAYLs					
	Cases (n), 1990	Cases (n), 2021	AAPC,1990–2021	p value	Cases (n), 1990		Cases (n), 2021		AAPC, 1990–2021	p value
Global	22,031 (16,236 to 28,461)	16,356 (12,828 to 20,343)	− 1.44 (− 1.57 to − 1.31)	< 0.001	1,863,538 (1,370,838–2,410,914)		1,371,309 (1,074,065 to 1,706,987)		− 1.46 (− 1.59 to − 1.33)	< 0.001
Sex										
Male	12,539 (7545 to 18,421)	8778 (5979 to 12,030)	− 1.66 (− 1.82 to − 1.49)	< 0.001	1,059,678 (635,296–1,561,078)		735,204 (499,629 to 1,007,972)		− 1.68 (− 1.84 to − 1.51)	< 0.001
Female	9492 (6226 to 14,929)	7578 (6242 to 8819)	− 1.22 (− 1.33 to − 1.13)	< 0.001	803,860 (525,064–1,271,695)		636,106 (523,007 to 741,090)		− 1.24 (− 1.36 to − 1.13)	< 0.001
Age group, years										
0–4	10,040 (7089 to 13,544)	5660 (4242 to 7405)	− 2.12 (− 2.26 to − 1.98)	< 0.001	888,845 (628,041–1,197,591)		502,601 (376,859 to 656,923)		− 2.11 (− 2.25 to − 1.97)	< 0.001
5–9	7235 (5447 to 9194)	5863 (4608 to 7130)	− 1.23 (− 1.38 to − 1.08)	< 0.001	603,360 (453,884–766,573)		490,210 (385,233 to 595,775)		− 1.22 (− 1.38 to − 1.07)	< 0.001
10–14	4756 (3701 to 5723)	4832 (3978 to 5808)	− 0.65 (− 0.77 to − 0.53)	< 0.001	371,333 (288,914–446,749)		378,499 (311,973 to 454,290)		− 0.64 (− 0.76 to − 0.52)	< 0.001

From 1990 to 2021, both the mortality and disability-adjusted life years (DALYs) for the disease consistently decreased (AAPC = − 1.44, 95% UI [− 1.57, − 1.31]; AAPC = − 1.46, 95% CI [− 1.59, − 1.33]). Joinpoint regression analysis revealed significant changes in mortality rates and DALYs from 1997 to 2018. As shown in Fig. 2 and Tables 1, 2.

### Age-standardized global trends by sex

Globally, the incidence of brain and CNS cancer has been declining in both boys and girls. The incidence rates decreased from 2.03 per 100,000 (95% UI [1.28, 2.87]) and 1.76 per 100,000 (95% UI [1.21, 2.58]) to 1.67 per 100,000 (95% UI [1.13, 2.27]) in boys and from 1.63 per 100,000 (95% UI [1.35, 1.91]) in girls. The average annual percentage change (AAPC) was − 0.68 (95% CI [− 0.82, − 0.53]) for boys and − 0.32 (95% CI [− 0.42, − 0.22]) for girls. Although there was a greater incidence in boys than in girls, the gap between the sexes gradually narrowed. From 1990 to 2021, there was an increasing trend in the prevalence of this disease in both boys and girls. The prevalence increased from 7.58 per 100,000 (95% UI [5.09, 10.38]) in boys and 6.96 per 100,000 (95% UI [5.02, 9.67]) in girls in 1990 to 8.17 per 100,000 (95% UI [5.56, 11.00]) in boys and 8.09 per 100,000 (95% UI [6.61, 9.55]) in girls by 2021. The AAPC for prevalence was 0.19 (95% CI [0.02, 0.35]) for boys and 0.38 (95% CI [0.25, 0.52]) for girls. As shown in Fig. S1 and Tables 1, 2.

Mortality rates and DALYs followed similar trends in both boys and girls, with a consistent decline. The AAPC for mortality was − 1.66 (95% CI [− 1.82, − 1.49]) for boys and − 1.22 (95% CI [− 1.33, − 1.13]) for girls. The AAPC regarding DALYs was − 1.68 (95% CI [− 1.84, − 1.51]) for boys and − 1.24 (95% CI [− 1.36, − 1.13]) for girls. As shown in Tables 1, 2.

### Global trends by age group

Globally, the incidence of brain and CNS cancer in children between the ages of 10 and 14 years increased from 1.36 per 100,000 (95% UI [1.10, 1.59]) in 1990 to 1.49 per 100,000 (95% UI [1.27, 1.80]) in 2021. The AAPC for this group was 0.29 (95% CI [0.14, 0.45]). In the other two age categories, the incidence exhibited a declining trend, with the most notable decrease observed in children under 5 years old, dropping from 2.47 per 100,000 (95% UI [1.81, 3.25]) in 1990 to 1.77 per 100,000 (95% UI [1.36, 2.25]) in 2021. The AAPC value was − 1.2 (95% CI [− 1.32, − 1.07]). In the 5–9 year age group, the AAPC was − 0.26 (95% CI [− 0.45, − 0.06]). As shown in Fig. S2 and Tables 1, 2.

The mortality and DALY trends were uniform across all three age categories, showing a declining trend. The most significant reduction in mortality occurred in children under 5 years of age, decreasing from 1.62 per 100,000 (95% UI [1.14, 2.18]) in 1990 to 0.86 per 100,000 (95% UI [0.64, 1.13]) in 2021, with an AAPC of − 2.12 (95% CI [− 2.26, − 1.98]). The AAPC values for the 5–9- and 10–14-year-old age categories were − 1.23 (95% CI [− 1.38, − 1.08]) and − 0.65 (95% CI [− 0.77, − 0.53]), respectively. Similarly, the most marked reduction in DALYs was found in children under 5 years of age. DALYs decreased from 143.38 (95% UI [101.31, 193.18]) in 1990 to 76.36 (95% UI [57.26, 99.81]) in 2021, with an AAPC of − 2.11 (95% CI [− 2.25, − 1.97]). The AAPC values for the 5–9- and 10–14-year-old age categories were − 1.22 (95% CI [− 1.38, − 1.07]) and − 0.64 (95% CI [− 0.76, − 0.52]), respectively. As shown in Fig. S2 and Tables 1, 2.

### Age-standardized global trends by SDI region

We divided the globe into five regions on the basis of the sociodemographic index (SDI) and compared childhood brain and CNS cancer incidence, mortality, and DALYs across these regions.

The incidence of childhood brain and CNS cancer was greater in the high- and upper-middle-SDI regions, whereas the low-SDI region had the lowest incidence. Overall, incidence rates declined in all regions, excluding the low- and lower-middle-SDI regions. The most notable decrease occurred in the middle-SDI region, where the incidence rate decreased from 2.22 per 100,000 (95% UI [1.56, 2.76]) in 1990 to 1.99 per 100,000 (95% UI [1.51, 2.53]) in 2021, with an AAPC of − 0.46 (95% CI [− 0.68, − 0.25]). In the upper-middle-SDI region, the AAPC was − 0.43 (95% CI [− 0.70, − 0.16]), whereas in the high-SDI region, it experienced only a mild reduction, with an AAPC of − 0.09 (95% CI [− 0.29, 0.10]). Notably, the incidence in the high-SDI region surpassed that in the upper-middle-SDI region for the first time in 2012. In terms of prevalence, the high-SDI region consistently had the highest rates. The prevalence increased across all regions, with the most notable increase in the upper-middle-SDI region, where it increased from 11.62 per 100,000 (95% UI [9.09, 14.11]) in 1990 to 16.34 per 100,000 (95% UI [12.74, 21.40]) in 2021, with an AAPC of 0.97 (95% CI [0.62, 1.32]). As shown in Fig. S3 and Tables 3, 4.

**Table 3:** The incidence and prevalence of childhood brain and central nervous system cancers and their AAPCs from 1990 to 2021 in five SDI regions

	Incidence				Prevalence			
	Cases (n), 1990	Cases (n), 2021	AAPC,1990–2021	p value	Cases (n), 1990	Cases (n), 2021	AAPC, 1990–2021	P value
High	5191 (4920 to 5477)	4751 (4352 to 5172)	− 0.09 (− 0.29 to 0.10)	0.351	29,869 (28,179 to 31,655)	32,338 (29,513 to 35,287)	0.47 (0.14 to 0.80)	0.005
Upper-middle	8201 (6390 to 9979)	6298 (4957 to 8149)	− 0.43 (− 0.70 to − 0.16)	0.002	31,518 (24,691 to 38,231)	37,338 (29,245 to 48,755)	0.97 (0.62 to 1.32)	< 0.001
Middle	12,792 (8959 to 15,882)	11,247 (8540 to 114,246)	− 0.46 (− 0.68 to − 0.25)	< 0.001	44,424 (30,943 to 55,336)	55,187 (41,287 to 70,926)	0.66 (0.42 to 0.91)	< 0.001
Lower-middle	5046 (3437 to 7807)	6890 (5226 to 8800)	0.40 (0.31 to 0.49)	< 0.001	15,688 (10,763 to 24,166)	25,370 (19,078 to 32,625)	0.95 (0.89 to 1.01)	< 0.001
Low	1851 (1093 to 3527)	3881 (2609 to 5212)	0.26 (0.05 to 0.47)	0.014	5543 (3262 to 10,675)	12,543 (8366 to 16,894)	0.50 (0.35 to 0.64)	< 0.001

**Table 4:** The mortality and DALYs of childhood brain and central nervous system cancers and their AAPCs from 1990 to 2021 in five SDI regions

	Mortality				DALYs			
	Cases (n), 1990	Cases (n), 2021	AAPC, 1990–2021	p value	Cases (n), 1990	Cases (n), 2021	AAPC, 1990–2021	p value
High	2007 (1918 to 2093)	1250 (1166 to 1342)	− 1.35 (− 1.56 to − 1.13)	< 0.001	168,640 (161,068 to 176,019)	105,002 (97,759 to 112,781)	− 1.34 (− 1.56 to − 1.12)	< 0.001
Upper-middle	5312 (4127 to 6468)	2237 (1830 to 2831)	− 2.36 (− 2.60 to − 2.11)	< 0.001	449,233 (347,823 to 547,131)	187,529 (153,181 to 237,547)	− 2.37 (− 2.62 to − 2.13)	< 0.001
Middle	9091 (6333 to 11,395)	5250 (4001 to 6553)	− 1.81 (− 1.99 to − 1.63)	< 0.001	769,696 (535,015 to 966,212)	438,456 (333,624 to 548,151)	− 1.85 (− 2.03 to − 1.66)	< 0.001
Lower-middle	4036 (2722 to 6340)	4602 (3528 to 5889)	− 0.20 (− 0.37 to − 0.02)	0.639	340,835 (229,650 to 537,485)	384,614 (294,364 to 492,550)	− 0.22 (− 0.39 to − 0.04)	0.592
Low	1566 (926 to 2998)	3004 (1989 to 4071)	− 0.06 (− 0.32 to 0.20)	0.027	133,629 (79,001 to 256,546)	254,615 (168,184 to 345,566)	− 0.07 (− 0.32 to 0.19)	0.014

Both mortality rates and DALYs significantly declined, particularly in the upper-middle-SDI region. Mortality rates decreased from 1.96 per 100,000 (95% UI [1.52, 2.38]) in 1990 to 0.96 per 100,000 (95% UI [0.79, 1.22]) in 2021, with an AAPC of − 2.36 (95% CI [− 2.60, − 2.11]). DALYs decreased from 165.74 (95% UI [128.14, 201.99]) to 81.25 (95% UI [66.16, 103.25]), with an AAPC of − 2.37 (95% CI [− 2.62, − 2.13]). As shown in Fig. S3 and Tables 3, 4.

### Regional age-standardized burden of childhood brain and CNS cancer

In 2021, East Asia had the greatest age-standardized incidence rate (ASIR) of childhood brain and CNS cancer, followed by the high-income Asia Pacific region. The greatest age-standardized prevalence rate (ASPR) was noted in high-income Asia Pacific, with high-income North America ranking second. Central Asia had the highest age-standardized rates of mortality (ASMRs) and DALYs, followed by Andean Latin America. Since 1990, the ASIR has increased in more than half of the regions, with the most notable increase occurring in southern sub-Saharan Africa. Prevalence rates have also increased in most regions, especially in North Africa and the Middle East. Mortality rates and DALYs have decreased in most regions, with the most significant reduction occurring in East Asia. Additionally, the ASIR, ASPR, and age-standardized rate of mortality.

**Fig. 3:**
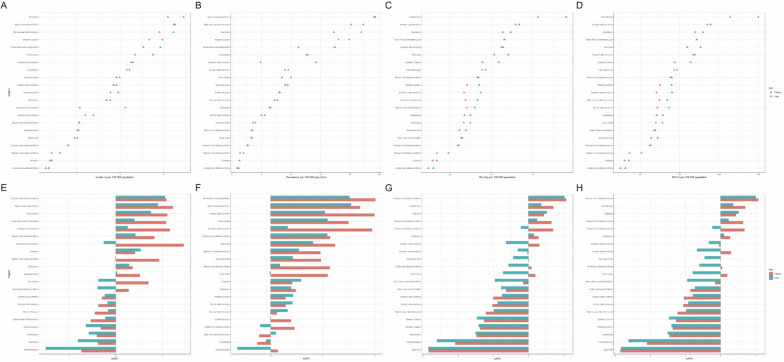
Regional age-standardized rates (per 100,000 population) of childhood brain and central nervous system cancers in 2021 and their percentage changes in rates for different sexes from 1990 to 2021. **A** Age-standardized incidence in 2021; **B** percentage change in age-standardized incidence, 1990–2021; **C** age-standardized prevalence in 2021; **D** percentage change in age-standardized prevalence, 1990–2021; **E** age-standardized mortality in 2021; **F** percentage change in age-standardized mortality, 1990–2021; **G** age-standardized DALYs in 2021; **H** percentage change in age-standardized DALYs, 1990–2021

ASMR and DALYs are generally higher for boys than for girls in most regions, which is consistent with global trends. As shown in Fig. 3.

**Fig. 4:**
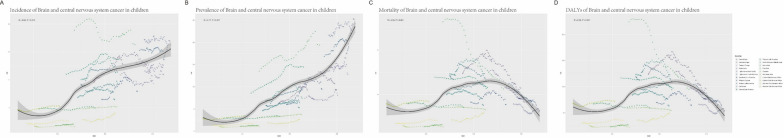
Trends for age-standardized rates (per 100,000 population) of childhood brain and central nervous system cancer among 21 regions by SDI from 1990 to 2021. **A** Age-standardized incidence; **B** age-standardized incidence; **C** age-standardized mortality; **D** age-standardized DALYs

The burden of childhood brain and CNS cancer varies significantly by SDI. Higher-SDI regions tend to have higher ASIRs and ASPRs. While the ASIR and ASPR generally increase with increasing SDI, regional patterns show considerable variation. Some regions exhibit declining ASIR and ASPR with increasing SDI, whereas other indices show increasing rates or no clear trend. Conversely, age-standardized death rates and DALYs consistently decrease with increasing SDI, with higher SDI areas experiencing lower ASMRs and DALYs. As shown in Fig. 4.

### National age-standardized trends

**Fig. 5:**
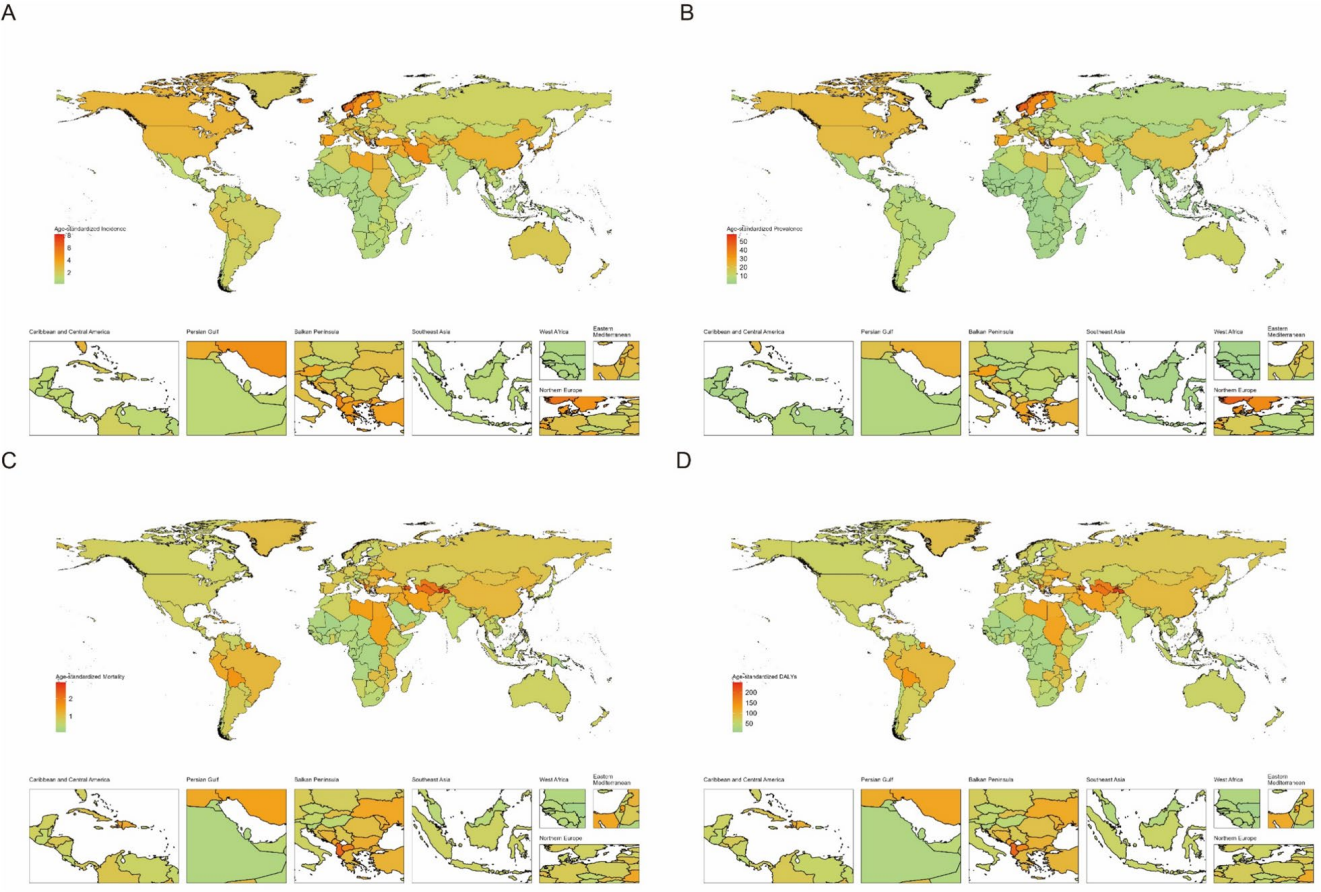
Countries’ age-standardized rates (per 100,000 population) of childhood brain and central nervous system cancers in 2021. **A** Age-standardized incidence; **B** age-standardized incidence; **C** age-standardized mortality; **D** age-standardized DALYs

In 2021, Monaco had the greatest ASIR of childhood brain and CNS cancer globally, with an incidence of 8.18 per 100,000 (95% UI [4.45, 13.41]). Conversely, Gambia had the lowest incidence at 0.15 per 100,000 (95% UI [0.07, 0.25]). Monaco also recorded the greatest ASPR at 58.25 per 100,000 (95% UI [31.61, 196.25]), whereas Gambia had the lowest prevalence rate of 0.49 per 100,000 (95% UI [0.22, 0.82]). As shown in Fig. 5.

For mortality rates and DALYs in 2021, Tajikistan reported the highest rates globally, with 2.93 per 100,000 (95% UI [1.46, 4.57]) for mortality and 249.10 (95% UI [123.31, 387.88]) for DALYs. In contrast, the Cook Islands had the lowest rates, with 0.11 per 100,000 (95% UI [0.06, 0.18]) for mortality and 8.98 (95% UI [4.79, 15.32]) for DALYs. As shown in Fig. 5.

**Fig. 6:**
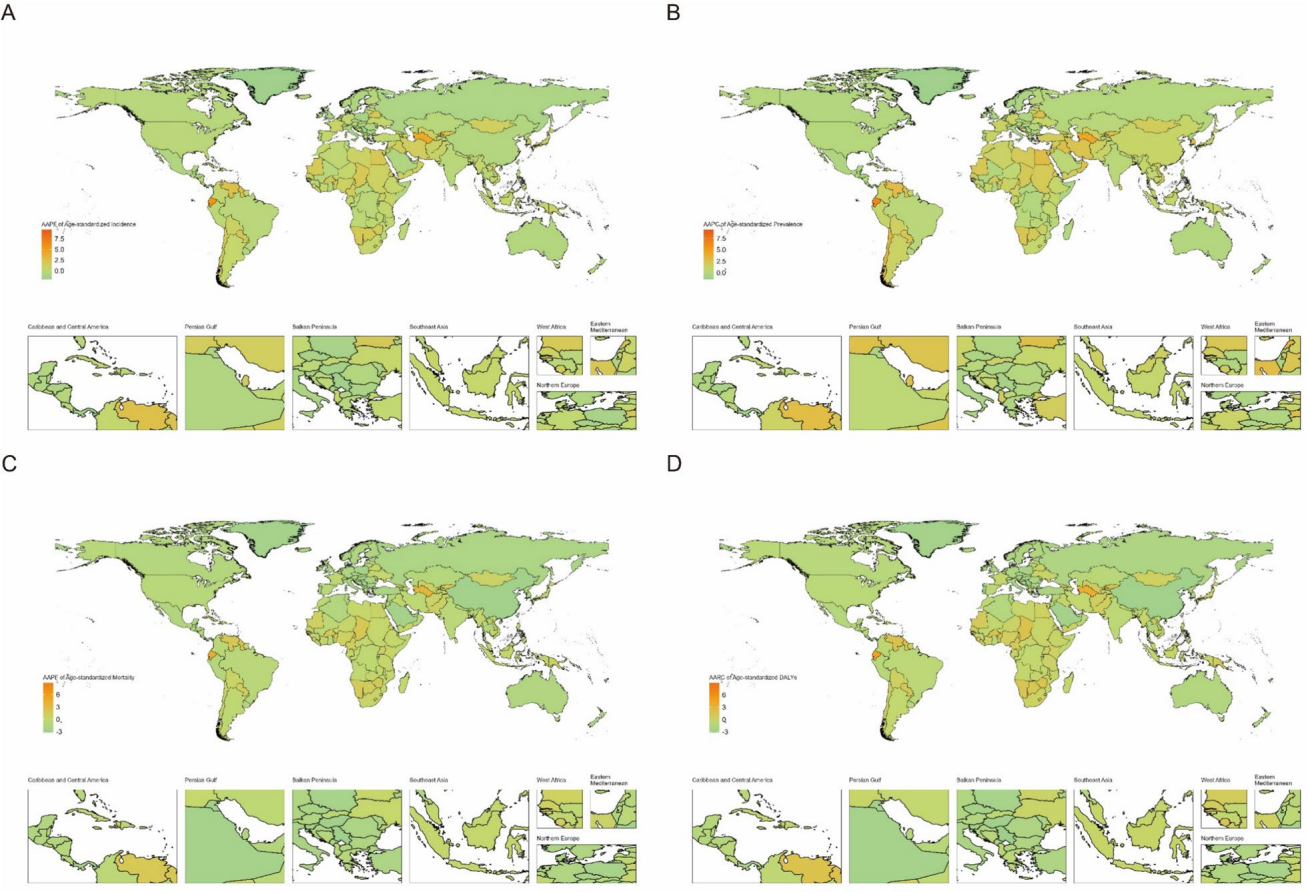
Trends for age-standardized rates (per 100,000 population) of childhood brain and central nervous system cancers in 204 countries from 1990 to 2021. **A** Age-standardized incidence; **B** age-standardized incidence; **C** age-standardized mortality; **D** age-standardized DALYs

Between 1990 and 2021, Greenland experienced the greatest decline in ASIR, with an AAPC of − 2.04 (95% CI [− 2.38, − 1.70]). Luxembourg observed the greatest decrease in ASPR, with an AAPC of − 2.41 (95% CI [− 4.74, − 0.02]). Luxembourg also had the largest reduction in the ASMR, with an AAPC of − 3.22 (95% CI [− 3.95, − 2.48]). Serbia experienced the greatest notable decline in DALYs, with an AAPC of − 3.17 (95% CI [− 3.69, − 2.64]). As shown in Fig. 6.

**Fig. 7:**
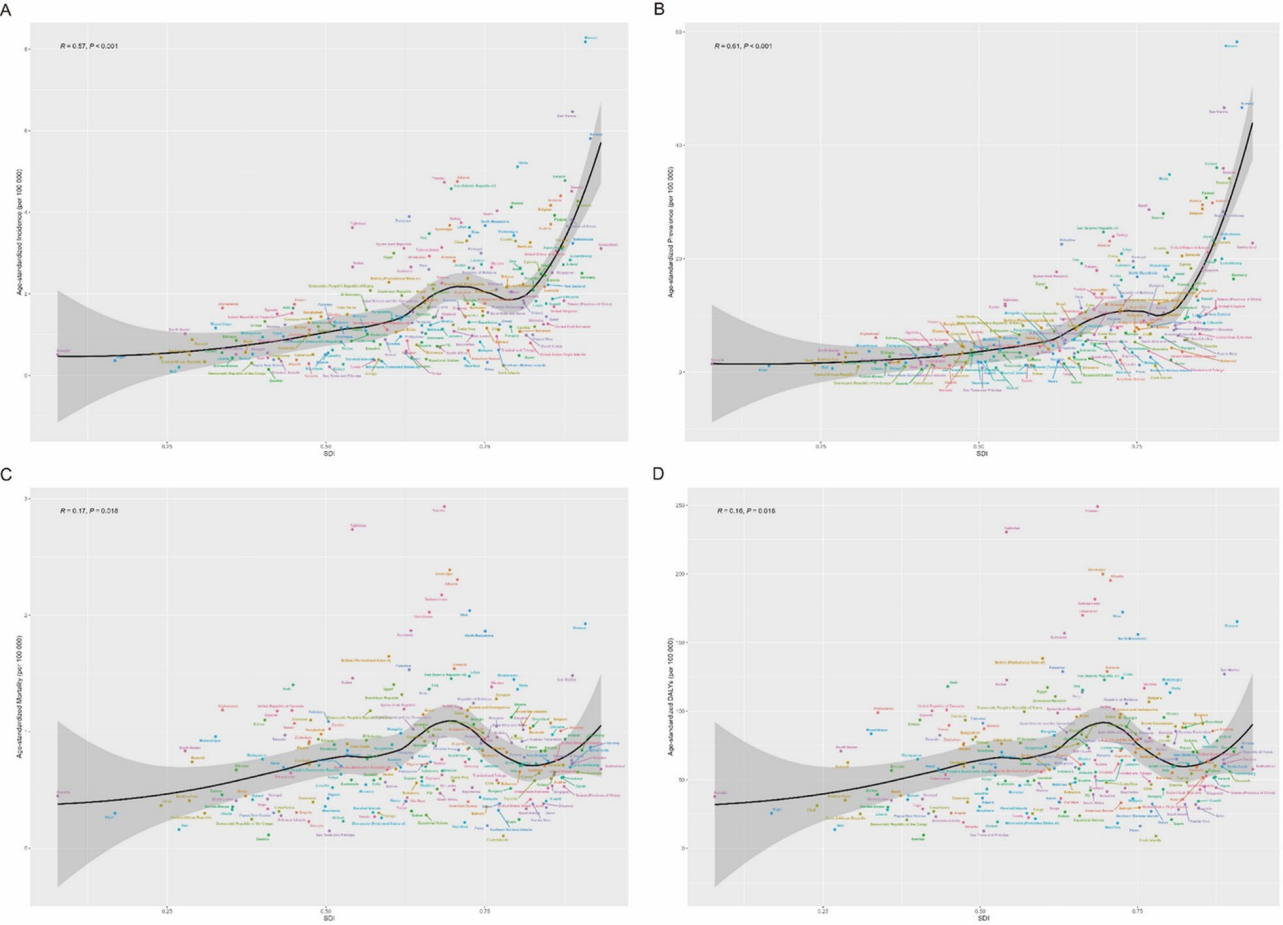
Trends for age-standardized rates (per 100,000 population) of childhood brain and central nervous system cancers among 204 countries with SDIs from 1990 to 2021. **A** Age-standardized incidence; **B** age-standardized incidence; **C** age-standardized mortality; **D** age-standardized DALYs

The burden of childhood brain and CNS cancer varies significantly on the basis of the SDI. ASIR and ASPR increase with increasing SDI values, clearly increasing. However, the ASMRs and DALYs are highest in regions with SDI values between 0.625 and 0.75. As shown in Fig. 7.

### Forecasting trends in childhood brain and CNS cancers

**Fig. 8:**
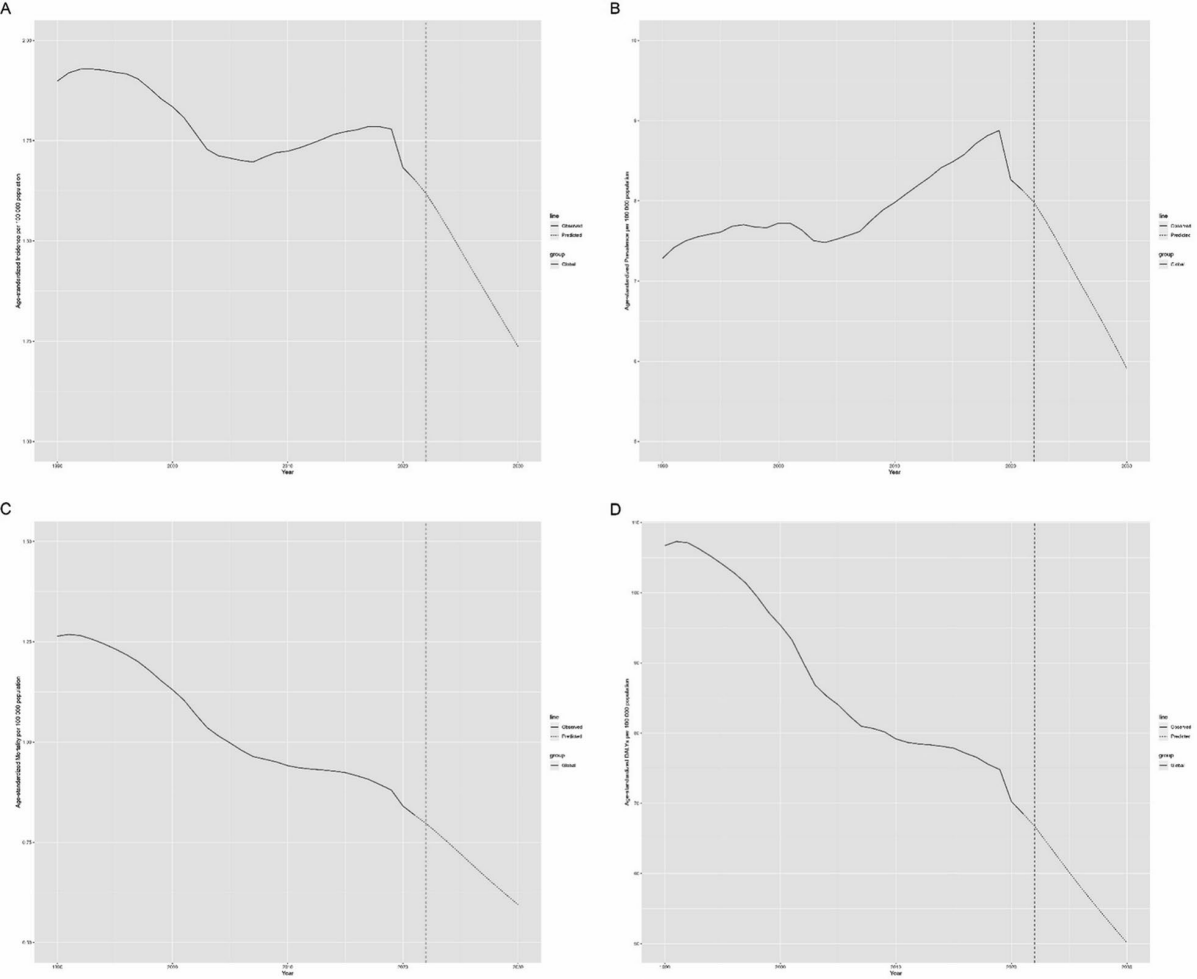
Prediction of change in the burden of childhood brain and central nervous system cancer to 2030. **A** Age-standardized incidence; **B** age-standardized incidence; **C** age-standardized mortality; **D** age-standardized DALYs

Figure 8 presents our forecast for future trends in childhood brain and CNS cancer. Our projections indicate a continued decline in all key metrics: incidence, prevalence, mortality, and DALYs. Specifically, the ASIR is expected to decrease from 1.65 per 100,000 in 2021 to 1.24 per 100,000 by 2030. ASPR is projected to decrease from 8.13 per 100,000 in 2021 to 5.91 per 100,000 in 2030. Similarly, the mortality rate is anticipated to decrease from 0.82 per 100,000 in 2021 to 0.59 per 100,000 in 2030. DALYs are forecasted to decrease from 68.54 in 2021 to 50.15 in 2030.

## Discussion

### Main interpretation

Childhood brain and CNS cancer is the most prevalent solid tumor in children aged 0–14 years [6], with incidence rates decreasing as children age and being higher in boys than in girls. The standard treatment for childhood gliomas typically involves complete surgical resection followed by radiotherapy, provided that it is feasible [18]. However, the high risks associated with craniotomy surgery, coupled with the toxic side effects of postoperative radiotherapy and chemotherapy, may lead to severe complications in pediatric patients, such as hemiplegia, epilepsy, and hydrocephalus. These conditions can significantly impact children's growth and development while imposing an immense burden on their families [9, 21, 22, 24]. These challenges contribute to the relatively rare occurrence of childhood brain and CNS cancers, which remain among the three most prominent causes of childhood mortality. Consequently, it poses a substantial global health burden [5, 7, 15].

The latest GBD study indicated that in 2021, there were 33,091 new cases of childhood brain and CNS cancer and 16,356 deaths worldwide. At present, there are 162,900 individuals with brain and CNS cancer and 1,371,300 disability-adjusted life years (DALYs) [23]. While the overall incidence of childhood brain and CNS cancer has been decreasing, there was a noticeable rebound between 2005 and 2019, as also reported by Miller et al. [25]. The increased application of CT imaging technology, the widespread adoption of MRI, the emergence of stereotactic biopsy techniques, and recent discoveries such as the DDX31-GFI1B and HAND2-MYC fusion proteins, coupled with innovations in cerebrospinal fluid diagnostic methods, have led to a resurgence in incidence rates [19, 26–28]. Previous research has indicated that brain and CNS cancer is most common in children aged 0–4 years, with the highest mortality rates occurring within the first year of life [5, 6, 13]. Recent advancements in prenatal diagnostic techniques and screening for tumor-susceptible syndromes have likely contributed to a decline in the incidence of this disease [6, 29]. In line with earlier studies, our findings show a consistent decline in global mortality and DALYs for childhood brain and CNS cancers from 1990 to 2021 [7]. This trend can be attributed to the standardization of treatment protocols, improved grading of treatment intensity, and advancements in therapies such as tumor vaccines, stem cell therapy and lysoviruses [17, 30, 31]. Stem cell therapy has made some progress in other neurological disorders, but its application in central nervous system tumors remains in the exploratory phase. Further research is warranted to reduce the mortality rates associated with these tumors [31, 32]. Our analysis of data from 1990 to 2021 revealed a notable acceleration in the rates of decline in incidence and mortality after 2018. This improvement is likely linked to the updated WHO classification of CNS cancers, as well as advancements in technologies such as fluorescence lifetime imaging microscopy, pediatric cancer model atlases, and proton therapy [16, 29, 33–37]. Our projections suggest that the incidence, mortality, and DALYs of this disease will continue to decrease in the future.

Significant differences were observed within the five sociodemographic index (SDI) regions, with high- and upper-high-SDI areas reporting higher rates of incidence, survival, mortality, and DALYs for childhood brain and CNS cancers than other regions did. The development of central nervous system tumors is associated with multiple factors. Genetic factors represent one of the significant risk factors, with approximately 5% of gliomas exhibiting familial occurrence. Longer leukocyte telomeres are associated with the development of glioma. Demographic factors, such as sex, ethnicity, and socioeconomic status, are also associated with tumor occurrence [38, 39]. Environmental factors such as ionizing radiation, carbon monoxide, butadiene, and automobile exhaust are also risk factors for tumor development [10, 40]. There are also other risk factors, such as diet and cytomegalovirus infection [41]. Moreover, regions with high SDIs benefit from more advanced healthcare systems and more sophisticated screening tools, which are factors that further influence cancer incidence rates. While the age-standardized rates of mortality (ASMRs) and DALYs are decreasing across all SDI regions, the lower availability of medical resources in lower-middle- and low-SDI regions leads to a slower decline in these metrics [7, 12]. The unequal distribution of healthcare resources across regions has led to this phenomenon. As emphasized by Uwishema and Boon (2025), strengthening healthcare infrastructure in low-SDI regions such as Africa is therefore essential [42]. Poor detection and management of complications during disease treatment also contribute to the slow decline in disease mortality rates in these low-SDI regions [22].

Among the 204 countries and regions analyzed, Africa has relatively low rates of incidence and high rates of mortality for childhood brain and CNS cancers. This is likely due to the continent's lower socioeconomic status [12]. Additionally, research indicates that black children have a lower incidence of brain and CNS cancers than white children do, possibly due to interethnic epigenetic differences [8, 43]. Underdiagnosis also contributes to the lower reported prevalence in Africa, with some studies showing that up to 50% of cases in low-income areas are underdiagnosed [20, 44]. Limited diagnostic capacity and barriers to healthcare access are key factors affecting disease diagnosis, which is consistent with a broader systematic review of brain cancer epidemiology in the region [20]. As mentioned by Uwishem et al., the healthcare systems in these regions have been impacted by events such as the COVID-19 pandemic, potentially leading to a further increase in the rate of missed diagnoses [45]. Furthermore, black children with brain and CNS cancers generally have higher mortality rates than white children do, which helps explain the elevated mortality rates reported in Western Asian countries. [43]

Unlike previous studies on childhood tumors, our research specifically examines brain and CNS cancers, incorporating the most recent data from 2020 and 2021. While we have made significant efforts to provide a thorough analysis, our study relies on secondary data from the GBD.

## Limitation

Our study is based on a secondary analysis of data from the Global Burden of Disease database. The accuracy of our data analysis depends on the accuracy of the original data. However, incomplete data inevitably exist during the disease data registration process, particularly in certain low-SDI regions. This leads to errors in the analysis results [20, 22]. Second, certain data are derived through statistical models, which may introduce bias between these data and the original data.

## Conclusion

This study offers a comprehensive view of the global burden of childhood brain and CNS cancers from various angles. Despite progress in medical science and technology leading to a reduction in disease burden, substantial human, material, and financial resources remain necessary to address this issue annually. We hope that this research will provide guidance for policymakers and encourage the effective allocation of global resources to further mitigate the impact of this disease.

## Novelty and impact

1. Analyzing the burden of brain and central nervous system cancer with the latest GBD data.
2. Multidimensional analysis of the burden of brain and central nervous system tumors in children.
3. Predicted trends in the disease burden of brain and central nervous system cancer in children.

## Supplementary Information

Additional file 1.

## Data Availability

All data used in this study can be find at the GBD 2021 study (https://vizhub.healthdata.org/gbd-results). In the meantime, we have uploaded the data used in this article to the supplementary material.

## Abbreviation

Brain and CNS cancer: Brain and central nervous system cancer
DALYs: Disability-adjusted life years
UI: Uncertainty interval
SDI: Sociodemographic index
GBD: Global Burden of Disease Study
AAPC: Average annual percentage change
APC: Annual percentage change
ASIR: Age-standardized incidence rate
ASPR: Age-standardized prevalence rate
ASMR: Age-standardized rate of mortality
