# Supplementary material for: Global, regional, and national childhood brain and central nervous system cancer burden: an analysis based on the Global Burden of Disease Study
Source: Trop Med Health. 2025 Oct 2;53:130. doi: 10.1186/s41182-025-00810-9 (PMC12492830; doi:10.1186/s41182-025-00810-9)
Supplement: Supplementary file 1 — Additional file 1. [file 41182_2025_810_MOESM1_ESM.docx]

**Supply Fig 1. Global trends in age-standardized incidence rates (per 100,000 population) of brain and central nervous system cancer in children by sex, 1990--2021.** (A) Age-standardized incidence rate; (B) age-standardized prevalence rate; (C) age-standardized death rate; (D) age-standardized DALYs.

**
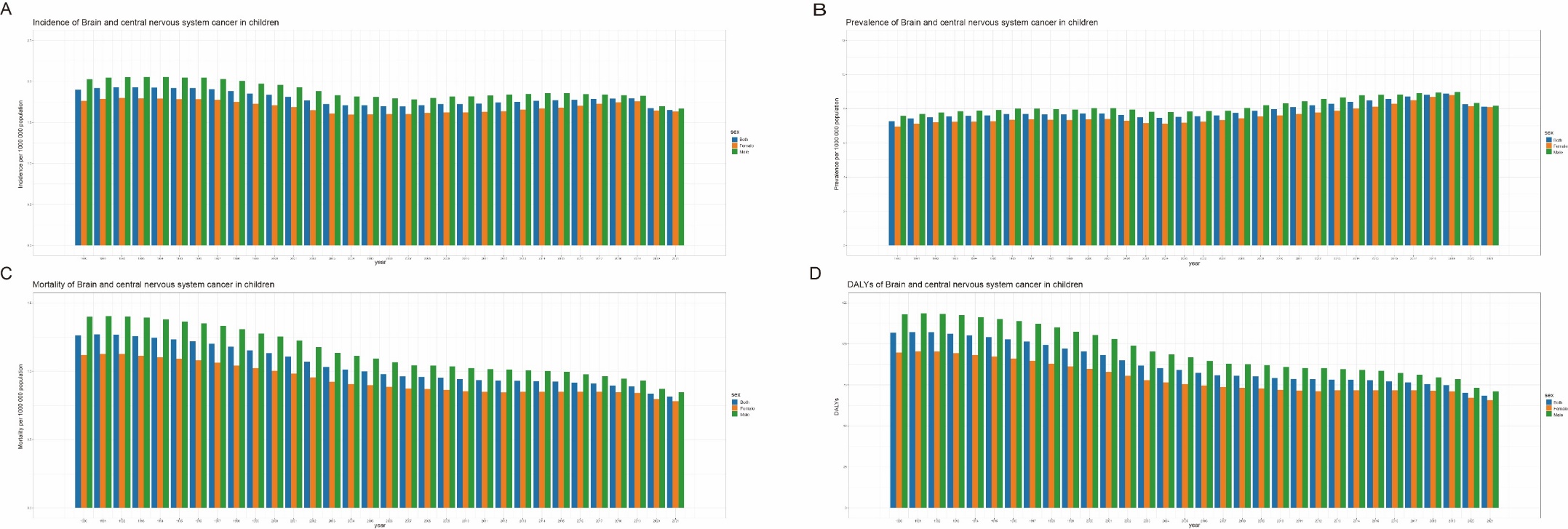
**

**Supply Fig 2.** **Global Burden of Disease for Cancer of the Brain and Central Nervous System in Children of Different Age Groups, 1990--2021.** (A) Incidence; (B) Prevalence; (C) Mortality; (D) DALYs

**
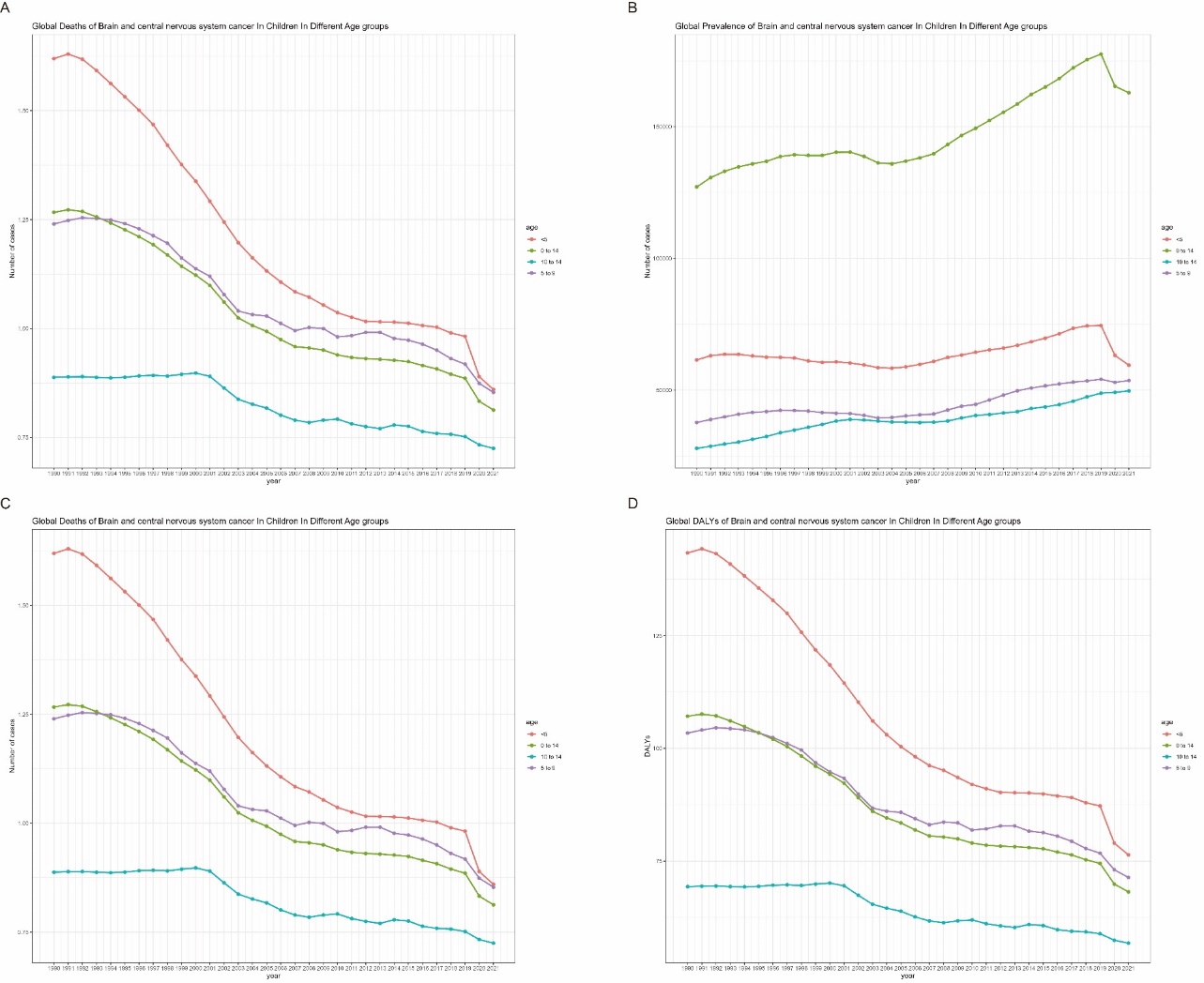
**

**Supply Fig 3.** **Global Burden of Disease for Childhood Cancer of the Brain and Central Nervous System in Different SDI Regions, 1990--2021.** (A) Age-standardized incidence rate; (B) age-standardized prevalence rate; (C) age-standardized death rate; (D) age-standardized DALYs.

**
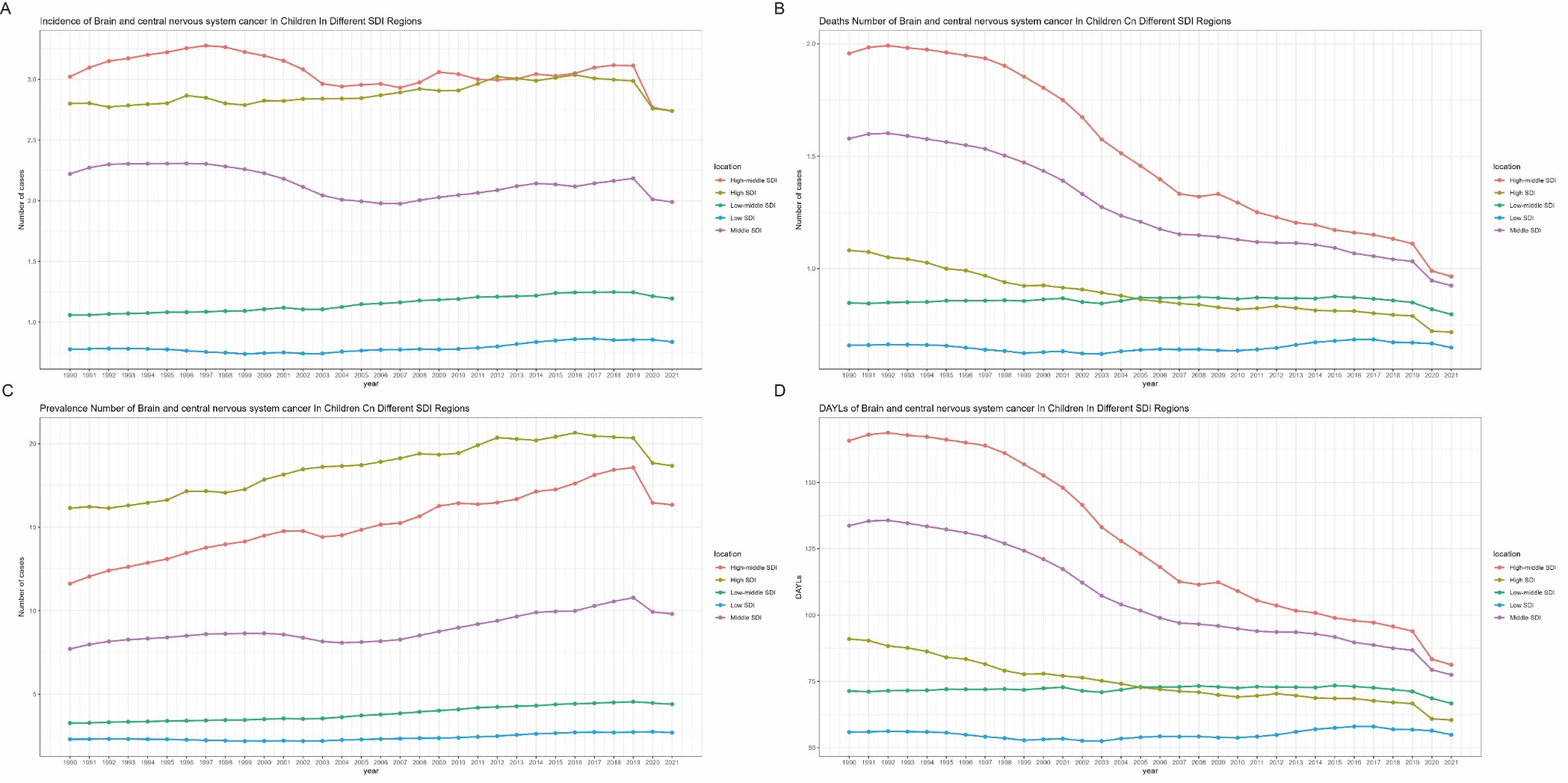
**
